# Description and Evaluation of a Pharmacy Graduate Health Services Research Methods Course

**DOI:** 10.3390/pharmacy12040127

**Published:** 2024-08-21

**Authors:** David R. Axon

**Affiliations:** Department of Pharmacy Practice & Science, R. Ken Coit College of Pharmacy, The University of Arizona, 1295 N. Martin Ave., Tucson, AZ 85721, USA; draxon@arizona.edu; Tel.: +1-520-621-9561

**Keywords:** pharmacy, course development, course evaluation, graduate education

## Abstract

The purpose of this paper is to provide a description and evaluation of a graduate-level Health Services Research Methods course offered at the University of Arizona R. Ken Coit College of Pharmacy. This three-credit, round-table discussion-style course introduces students to fundamental concepts in healthcare study design and teaches them how to design and critique example studies for a variety of commonly encountered study designs. The course is assessed through essay-style examinations, development of a research proposal, and low-stakes weekly assignments. Twenty-seven students have completed the course in the past five years. Feedback from student course surveys was almost unanimously positive, with few meaningful suggestions for improvement. The description and evaluation of a graduate-level Health Services Research Methods course at one institution indicates that students had a largely favorable experience with the course. Considerations for future revisions to the course are discussed alongside other lessons learned.

## 1. Introduction

There exists a body of published literature on the development, evaluation, and faculty reflections of Doctor of Pharmacy (PharmD) curricula and coursework in the fields of study design and statistics [1,2,3,4]. There is also literature available for similar healthcare professionals, such as student or resident physicians [5,6] and others [7]. These articles describe the content and outcomes of these courses in their respective contexts. However, there is little documentation offering insights into the design and evaluation of graduate programs offered by colleges of pharmacy [8]. One example of such a graduate program is the field of Social and Administrative Pharmacy (SAP). Several SAP programs exist across the United States, and many have evolved over time to focus on specific areas of interest, such as Health Economics and Outcomes Research (HEOR) and Health Services Research (HSR).

At the University of Arizona R. Ken Coit College of Pharmacy, the latest incarnation of this program is known as the Health and Pharmaceutical Outcomes graduate program, which offers Masters (MS) and Doctor of Philosophy (PhD) degrees [9]. Students in this program are required to complete a series of required courses that include topics such as biostatistics, epidemiology, study design, health technology assessment, and health policy, alongside the student’s choice of elective coursework, independent study credits, and their thesis or dissertation research.

The purpose of this paper is to provide a description of one course, the Health Services Research Methods (HSRM) course, offered as part of the University of Arizona Health and Pharmaceutical Outcomes graduate program coursework. This is presented alongside a brief synopsis of student feedback and performance as well as lessons learned from the course director from teaching this course over the past five years. It is hoped that this paper will offer some insight into pharmacy graduate program courses and offer inspiration for those who are seeking to develop or redesign a similar course at their institution.

## 2. Description of the Course

The HSRM graduate course is required for MS and PhD students and is typically offered in-person in the Fall semester of their first year of study. The course usually attracts graduate students from other programs such as medicine, public health, and business, and occasionally includes a student pharmacist taking the course as part of their pharmacy program elective requirements. The HSRM course was originally developed to aid student development and understanding of study designs commonly encountered in HEOR or HSR. The HSRM course is three credit units and meets for three hours every week over the 15-week semester, with an expectation that students spend an additional six hours per week on average outside of class on self-study. These expectations are consistent with credit unit guidance at the author’s institution. The course has 10 learning objectives which are outlined in Table 1. These objectives (and subsequent course content) were developed by the program faculty based on their expertise of knowledge and skills students need to acquire during their studies.

The course was historically taught by a panel of faculty, each contributing their expertise, although in recent years, due to faculty departures, the course has been taught by just one faculty member (the author of this paper). Early weeks of the course commonly involve a lecture covering foundational concepts, while later weeks typically involve a round-table discussion of study designs with worked examples. This approach was chosen so that the instructor could ensure knowledge of foundational concepts were imparted to students with opportunities to discuss as necessary, before applying these concepts in the study designs covered in later weeks. The round-table discussion style of the course was deemed appropriate for a small group of students covering complex topics, so that they could prepare the materials at their own pace outside of class and share knowledge, experiences, and questions during class. Students are expected to complete pre-class activities, which often include review of published papers exemplifying a particular study design and worksheets or other homework assignments, to initiate their critical thinking and be prepared for a shared discussion during class time. This allowed students to share knowledge and seek clarification from their peers and the instructor.

The course is supported by online course management software (currently Desire 2 Learn (D2L) [10]) that contains a folder with the documents required for each week. In each folder, there is a one-page instruction document that provides the learning objectives, required and suggested readings, and any pre-class homework assignments. The exact course content and activities have evolved over time to remain contemporary and meet the needs of students [11] but have typically included the topics outlined in Table 2.

During the COVID-19 pandemic, the course was taught in an online, synchronous format. The content and activities remained the same, but the lecture and discussion sections occurred online instead of in-person.

The introduction to the scientific method module provides students with an understanding of what research is and is not and explains the process of scientific inquiry and the principles of evidence-based medicine. The ethics in research and human subjects protection module provides students with an understanding of historical events that led to the need for regulations, a brief overview of the Belmont report [12] that includes respect for persons, beneficence, and justice, appreciation of the Common Rule [13], and the role of an Institutional Review Board, as well as self-study and group discussion on a local case where the principles of the Belmont report were not followed and the consequences that resulted from this [14]. Students also complete the Collaborative Institutional Training Initiative (CITI) program [15] so that they are prepared to undertake research as part of their graduate studies.

In the study proposal module, students are introduced to the basic principles of preparing a research proposal and review an example proposal [16]. As part of the course assessment, students are expected to prepare, present, and submit a research proposal for a study of their choice (see later). In the literature searching module, students meet with a health sciences librarian who introduces them to the many library resources that are available to them, as well as how to search for relevant literature through databases (e.g., PubMed) and manage their references using citation management software (e.g., Endnote v.21). The next three modules (reliability and validity, biostatistics and epidemiology, sampling strategies and introduction to study designs) are typically lecture-based and are designed to introduce students to the fundamental concepts that will be needed to understand and discuss the study designs that will be covered in subsequent weeks of the course.

Weeks 6 to 14 involve the discussion of worked examples of commonly encountered study designs, typically focusing on two or three related examples per week. This begins with an examination of true experimental designs such as randomized controlled trials (RCTs) and related designs such as crossover studies. Subsequent weeks explore observational designs such as prospective and retrospective cohort studies, case–control studies, cross-sectional studies, pretest-posttest designs, case reports, and descriptive studies. Different types of literature reviews and an overview of meta-analysis are then covered, while qualitative designs, survey designs, and pharmacoeconomic designs complete the course content.

In the final week of the course, students present their research project proposals (introduced at the start of the course) and receive feedback from peers and the course instructor. Any remaining course time is dedicated to seeking student feedback on the course and an opportunity for students to ask any questions or review any difficult concepts again.

## 3. Course Assessment

There are three main aspects of assessment in this course. The first is a take-home examination at the end of the semester. This typically includes five essay-style questions covering the major study designs taught in the course and is worth 50% of the course grade. An example examination question may be to design an RCT from scratch or critique a published RCT that is provided to them.

The second assessment is the development and presentation of a research proposal for a study that they would like to complete and is worth 30% of the course grade. Students are introduced to the concept of research proposals early in the course and given an example template on which to develop their own study (see earlier). Students can develop their study throughout the semester using a study design of interest to them and may seek feedback from the course instructor during or outside of class. Typically, there is spare time in class throughout the semester where students have an opportunity to provide an update on their research proposals and ask any questions they may have. Students are encouraged to develop a research proposal for a project they would like to carry out, and many of them do complete their proposed project for independent study the following semester. This activity is designed to help prepare students for independent research study and, later, their MS or PhD dissertation/thesis.

The final 20% of the course grade is for low-stakes assignments throughout the course including weekly completion of pre-class homework activities and in-class participation.

## 4. Findings

Enrollment in the course has ranged from five to seven students per year, for a total of twenty-seven students over the past five years. Of these, 24 students earned an A grade, with 1 student each earning a B grade, C, grade, or fail grade. The average grade for the course is therefore an A grade. The failing student did not wish to pursue course remediation. Findings from this course description and evaluation paper are limited to a summary of information obtained from student course surveys administered by the University at the end of each course. The student course survey consistently asks 12 questions about the course on a five-point scale (strongly agree, agree, uncertain, disagree, strongly disagree), along with open-response questions seeking comments on what the students liked about the course and suggestions to improve the course.

Over the past five years, 23 of the 27 students submitted the student course survey. For almost all years and questions, students indicated a response of strongly agree or agree, except for two uncertain and two disagree responses during the COVID-19 pandemic when the course was taught online. Comments received from students regarding what they liked about the course commonly mentioned the organization of the course, the materials and activities provided, the small-group, round-table discussion style of the course, the ability to share knowledge and experience among the group, and the knowledge and teaching style of the instructor, which are described below:

Organization of the course: Students commented that the contents provided were well defined, and they appreciated how well-organized the topics for each class were.

Materials and activities provided: Students commented that they liked evaluating the different research papers provided and found the materials and examples informative. Other students commented that the content was detailed, clear, concise, and easy to understand.

Small-group, round-table discussion style of the course: Students commented that they enjoyed the discussion of each topic and the “flipped” classroom-style approach. They said the approach of reading and completing tasks ahead of class and then discussing in class was an effective learning approach. One mentioned that it could be hard to do this but it was a worthy learning experience. Students enjoyed being able to share ideas and collaborate with their peers in class.

Ability to share knowledge and experience among the group: Students commented that the open, seminar style of the course encouraged everyone to contribute, and they enjoyed being able to discuss assignments with peers.

Knowledge and teaching style of the instructor: Students appreciated the knowledge and passion of the instructor, as well as the encouraging and fostering nature of the instructor. Students appreciated the instructors’ teaching expertise and style of delivery as well as promptness responding to queries outside of class. Students commented that the instructor was able to present complex topics in an easy-to-understand way, with no judgment towards those who needed additional explanation.

Meaningful suggestions from students to improve the course were rarely provided but included suggestions for additional materials (particularly for topics that were more complex, e.g., case–control study designs), more sharing of knowledge between students (particularly those with more experience than them), and the opportunity to conduct a group project as part of the class. After experiences with online learning during COVID-19 pandemic, there were also a few comments around the desire for an online or hybrid course, the ability to attend class remotely, and splitting the three-hour weekly class time into smaller segments.

## 5. Discussion

The discussion of this paper is focused on the author’s experience and lessons learned teaching this class over the past five years, as well as reflection from student feedback.

The first lesson learned is the need for regular evolution of the content taught in the course to make sure it remains up-to-date for the needs of students in an era where methodological approaches are rapidly evolving. This course was designed to teach the fundamental concepts of study design to students, which remain important regardless of methodological advances. As ever, it is a difficult balance to retain existing material that remains important while adding new material without overburdening students relative to the course credit received. One simple solution to this problem is to expand the number of course credits offered, while an alternative solution is to create a second course that is taught in the following semester (e.g., Advanced Health Service Research Methods). This second course could include more advanced topics, a discussion of new methodologies that build on the foundational topics already studied, as well as an opportunity to conduct the research project proposed in the previous semester. For instance, understanding the advances in real-world data/evidence studies and learning how to appropriately use artificial intelligence in the design and conduct of research studies are topics that could be included in such a course. Other ideas include an applied HSRM course, where students apply their knowledge of study design to develop their own studies. Additional course topics could be introduced from the recently compiled compendium of contemporary health services research methods literature [17].

A second consideration is how to offer this course in a format that meets students demands for alternative or more flexible modes of learning. This request has been more apparent since the COVID-19 pandemic, when many students experienced online or remote education. For example, while some students prefer an in-person course for the regular scheduling and opportunity to interact with the instructor and peers in-person, others would like the flexibility to study the material asynchronously at a time of their choosing or be able to choose whether they attend class in-person or online. This conundrum presents an opportunity for growth of the graduate program and expanded educational opportunities for students, while at the same time presenting challenges for universities and instructors with faculty workload and ensuring a consistent and comparable learning experience regardless of course modality. In the case of this HSRM course that uses a small-group discussion format, a live online or hybrid option may be appropriate, but it would be difficult to translate this course into an asynchronous online version that retains other aspects of the course that students enjoyed, such as the ability to discuss materials with their peers. Instead, it would be better to develop a reimagined course more suited to the online experience. However, this requires resources (e.g., faculty time) and raises questions of equivalence between the two course modalities. Recent literature has suggested that students appreciate having a choice of course modality but that their decisions on which approach works best for them is more complex than previously thought [18]. Previous work has described a flipped classroom approach for an online public health graduate course, which found that students had a positive learning experience while developing their critical thinking skills and being able to interact meaningfully with their peers [19]. Regardless of approach, the literature suggests students gain a high level of understanding the course content, experience good grades, and are equally satisfied [20,21].

Further considerations are the faculty available to teach the course and any alternative modalities of the course developed in future. As already noted, this course was originally taught by several faculty members but is now taught by only one faculty member. One of the benefits of having one faculty member teach the entire course is the consistency in learning style and expectations, flexibility if schedules must be moved or additional time is needed for certain topics, and the ability to easily link different concepts taught throughout the course. Challenges and limitations of this approach include faculty workload considerations, scheduling conflicts (e.g., if the faculty member has to attend to research or travel commitments), as well as limited opportunities for students to meet and learn from the expertise of other faculty members, particularly those who may have familiarity with novel methodologies. Specialist courses such as this also require contingency planning from administrators, given that the course would not be able to run if the only faculty member teaching the course became unavailable. Course co-directors or additional faculty could be added (based on available resources) to help reduce this risk.

Finally, it should be noted that this paper contains the perspective of one author teaching one course at one institution during a turbulent few years (e.g., the COVID-19 pandemic), and hence the data and opinions presented may not be representative or generalizable beyond this one course. In addition, the nature of this specialized graduate course resulted in a small data sample. Although the students provided a largely favorable ratings of the course, it is possible that students are reluctant to give poor ratings or do not take the time to provide a thoughtful critique of the course to help with future improvement. However, there may be some lessons learned that could be valuable for others to digest and consider when developing or evaluating courses at their own institutions. There may be value in encouraging instructors at other institutions to disseminate a discussion of the design and evaluation of their graduate courses, with the intent of building up repertoire of resources for the academic community to draw upon.

## 6. Conclusions

This paper provides a description of a graduate-level HSRM course at one institution. Evaluation of the course based on student course surveys over the past five years suggests that students had a largely favorable experience with the course. Considerations for future revisions to the course are discussed alongside other lessons learned. Additional publications of courses at other institutions are welcome to better understand the design and evaluation of graduate courses.

## Figures and Tables

**Table 1:** Health Services Research Methods course objectives.

By the End of This Course the Student Will Be Able to:
Describe the ethical considerations in health services research.
Discuss the scientific method.
Identify the logical structure of a research report.
Compare and contrast experimental research designs to other research designs.
List and describe the threats to the internal and external validity of study designs.
Identify the main sampling techniques and discuss the major advantages and disadvantages of each.
Discuss the primary advantages and disadvantages of the main data collection modes in survey research, particularly telephone interviews, face-to-face interviews, and mailed questionnaires.
Apply the principles of research to the testing of hypotheses in the various research designs.
Recognize how threats to internal validity are addressed in a research report.
Complete a protocol/proposal for conducting a research study.

**Table 2:** Health Services Research Methods topics taught in a typical iteration of the course.

Class	Topic(s)
1	Course overview, scientific method, ethics, and human subjects protection
2	Study proposals and literature searching
3	Reliability and validity
4	Biostatistics and epidemiology
5	Sampling strategies and introduction to study designs
6	True experimental design (randomized controlled trials) and crossover design
7	Cohort (prospective and retrospective) design and case–control design
8	Pretest-posttest design, before-after design, and cross-sectional design
9	Case-report/series and descriptive design
10	Literature reviews and meta-analysis
11	Survey designs 1
12	Survey designs 2
13	Qualitative research designs
14	Pharmacoeconomic designs
15	Proposal presentations and course review

## Data Availability

No new data were created or analyzed in this study. Data sharing is not applicable to this article.
